# Characterization and Calculation of the Dynamic Recrystallization Texture in Fe-3.0 Wt.% Si Alloy

**DOI:** 10.3390/ma15020517

**Published:** 2022-01-10

**Authors:** Guangshuai Shao, Yuhui Sha, Xi Chen, Songtao Chang, Fang Zhang, Liang Zuo

**Affiliations:** Key Laboratory for Anisotropy and Texture of Materials, Ministry of Education, Northeastern University, Shenyang 110819, China; guangshuaishao@126.com (G.S.); chenxineu@163.com (X.C.); Cst_Chang@163.com (S.C.); zhangf@smm.neu.edu.cn (F.Z.); lzuo@mail.neu.edu.cn (L.Z.)

**Keywords:** texture, dynamic recrystallization, strain-induced boundary migration (SIBM), Taylor factor, silicon steel

## Abstract

High-temperature plane-strain compression tests were performed on Fe-3.0 wt.% Si alloy from 900 °C to 1150 °C at strain rates of 5 s^−1^ to 1 s^−1^, and the texture development from different initial textures was investigated by means of electron backscattered diffraction. Dynamic recrystallization occurs by strain-induced boundary migration, and the evolutions of the microstructure and different texture components vary with the initial texture. The critical orientation boundary separating the weakened and enhanced texture components moves with the initial texture, and a quantitative relationship is established to represent the dependence of the critical Taylor factor on the instantaneous texture. A model is proposed to describe the dynamic recrystallization texture by incorporating the oriented nucleation probability with a variable critical Taylor factor. The present work could improve the accuracy of hot deformation texture prediction based on strain-induced boundary migration.

## 1. Introduction

The hot deformation texture exerts a significant influence on subsequent textural development through cold deformation and annealing processes. The textural evolution during hot deformation results from orientation rotation by plastic deformation [1,2], and is accompanied by dynamic recrystallization (DRX), which is related to the initial texture and deformation parameters. In the severe deformation of low- to medium-stacking fault energy (SFE) materials with a large stored strain energy gradient, DRX usually occurs by discontinuous dynamic recrystallization, where new dislocation-free grains nucleate from deformed grains and subsequently grow [3,4,5]. However, at a high deformation temperatures and low strain rate of medium- to high-SFE materials without a large stored strain energy gradient, DRX by continuous dynamic recrystallization (CDRX) and strain-induced boundary migration (SIBM) will take place, and the kinetic advantage determines the mechanism which plays the dominant role. CDRX is characterized by subgrain formation and growth, and the nucleation orientation may be low/high in stored strain energy [5,6,7]. Baczynski reported that CDRX with low stored strain energy dominates the nucleation of Ti-IF steel [8]. SIBM involves the bulges of the pre-existing grain boundary, and some DRX grains are closed with the formation of large-angle sub-boundaries, whereas some are not [9,10]. The bulging characteristic indicates the advantage of SIBM over CDRX, and the growth of DRX grains is assisted by SIBM. Texture evolution by SIBM is generally characteristic of the enhancing of textures with low stored strain energy, and the weakening of those with high stored strain energy [9,10,11].

Texture evolution due to SIBM has been observed to correlate with the initial texture in terms of Taylor factor distribution [12,13]. In the plane-strain compression of Ni-30 wt.% Fe alloy [14,15], {001}<100> increases slightly and {110}<110> changes little for the initial texture, mainly comprising {013}<100> with Taylor factor 2.4. In contrast, {001}<100> increases and {110}<110> reduces greatly for initial textures with a Taylor factor between 2.1 and 4.3. In the torsion of aluminium [16] and Ni-30 wt.% Fe alloy [10], {111}<112> and {112}<110> have a slight change for an initial weak shear texture consisting mainly of {111}<112> with Taylor factor 2, whereas {111}<112> increases greatly and {112}<110> remains stable for the initial random texture. In the compression of Fe-3.0 wt.% Si alloy [17,18], {001}<110> increases and the γ fibre (<111>//ND, normal direction) decreases in the initial random texture, while {001}<110> increases and the γ fibre decreases greatly for an initial texture mainly comprising {110}<110> with a Taylor factor of 4.3 and a λ fibre (<001>//ND) with a Taylor factor between 2.1 and 2.5.

Kestens [19] and Sidor [20,21,22] proposed an SIBM model in which low-Taylor-factor components nucleate with a constant probability. Baczynski and Jonas [8] suggested that the nucleation probability depends on both the critical and minimum Taylor factors. The published research is focused on an estimated constant critical Taylor factor, indicating that the Taylor factor range undergoes no change. In actuality, various texture components can show distinct evolution kinetics and even an opposite tendency, such that the Taylor factor range changes continuously during hot deformation. However, texture evolution by SIBM has not yet been clearly described with the different initial textures under consideration.

In the present work, the hot deformation texture in Fe-3.0 wt.% Si alloy was investigated by plane-strain compression for different initial textures. A quantitative relationship was established to represent the dependence of the critical Taylor factor on the instantaneous texture, and a model was proposed to capture accurately the texture evolution due to SIBM by introducing the continuous variation of the Taylor factor distribution associated with the initial texture and DRX process.

## 2. Materials and Methods

**Figure 1:**
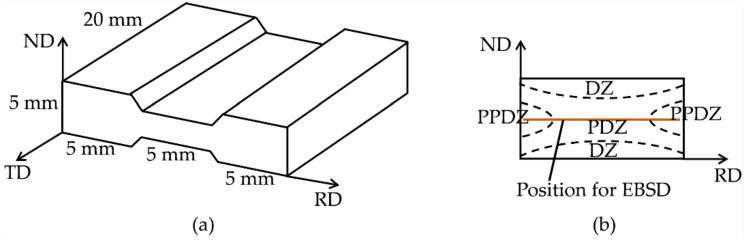
(**a**) The geometry of the plane-strain specimen, (**b**) diagram of the characteristic region of the effective strain distribution in the RD-ND section of the deformation zone.

Fe-3.0 wt.% Si sheets—which contained 0.003 wt.% C, 3.0 wt.% Si, 0.02 wt.% Mn, 0.001 wt.% S, 0.019 wt.% P, and balance Fe—were prepared by four different mechanical processes. In order to obtain Fe-3.0 wt.% Si specimens with the same average grain size but different initial textures, the sheets were annealed at 1150 °C × 10~25 min. Fe-3.0 wt.% Si specimens with four different initial textures were cut from the sheets for plane-strain compression on an MMS-200 thermo-simulation machine (State Key Laboratory of Rolling and Automation of Northeastern University, Shenyang, People’s Republic of China). The specimen geometry in reference to rolling is described in Figure 1a, and a diagram of the characteristic region of effective strain distribution in the RD-ND section of the deformation zone is shown in Figure 1b. The deformation zone of a specimen can be divided into three parts: the plastic deformation zone (PDZ) in the center, the partial plastic deformation zone (PPDZ) in the lateral sides, and the dead zone (DZ) at the top and bottom regions of the contact surfaces. The specimens were first heated at 10 °C·s^−1^ to the target deformation temperature, and then held for 1 min to eliminate the in-specimen temperature gradient. Compression testing was carried out at 1150 °C with a strain rate of 1 s^−1^, as well as 900 °C with 5 s^−1^, and the specimens were water-quenched immediately after being compressed to 50% reduction.

The DRX fraction in the hot compressed specimens was determined by the total volume fraction increments of low-Taylor-factor texture components. The microstructure and texture were measured using the electron backscattered diffraction (EBSD) technique (JEOL, Tokyo, Japan). EBSD samples were electro-polished under a voltage of 20 V for 15 s in a solution of 92% ethanol and 8% perchloric acid. The EBSD tests were performed in a field emission gun scanning electron microscope (FEG-SEM, JEOL JSM-7001F, Tokyo, Japan) with an electron accelerating voltage of 20 kV at a working distance of 15 mm. The EBSD measurement was performed on the center region of a specimen consisting of PDZ and PPDZ to avoid the potential influence of the position, which was marked in Figure 1b. The EBSD data was analyzed with HKL Channel 5 software.

## 3. Results

### 3.1. Microstructure Evolution

**Figure 2:**
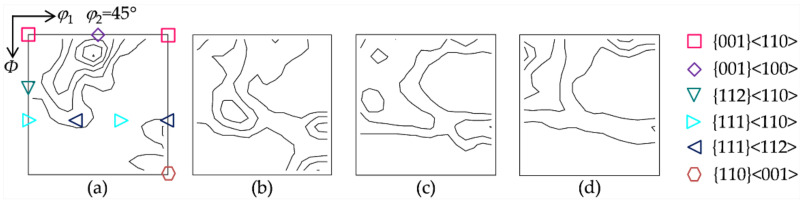
Constant *φ*_2_ = 45° section of the ODFs (levels: 1, 2, 3…) for the four initial textures: (**a**) initial texture I, (**b**) initial texture II, (**c**) initial texture III, and (**d**) initial texture IV.

Figure 2 illustrates the four initial textures prepared for hot deformation. Initial texture I gives the deviated {001}<100> extending to {113}<361>, as well as minor {111}<112> and {110}<001>. Initial texture II consists of strong {110}<001>, γ fiber with a peak at {111}<112>, and weak λ and α fibers (<110>//RD, rolling direction). Initial texture III exhibits a similar intensity along the λ, α and γ fibers. Initial texture IV is composed of λ fiber with a peak at {001}<210> and partial α fiber with a peak at {114}<110>, together with weak γ fiber.

**Figure 3:**
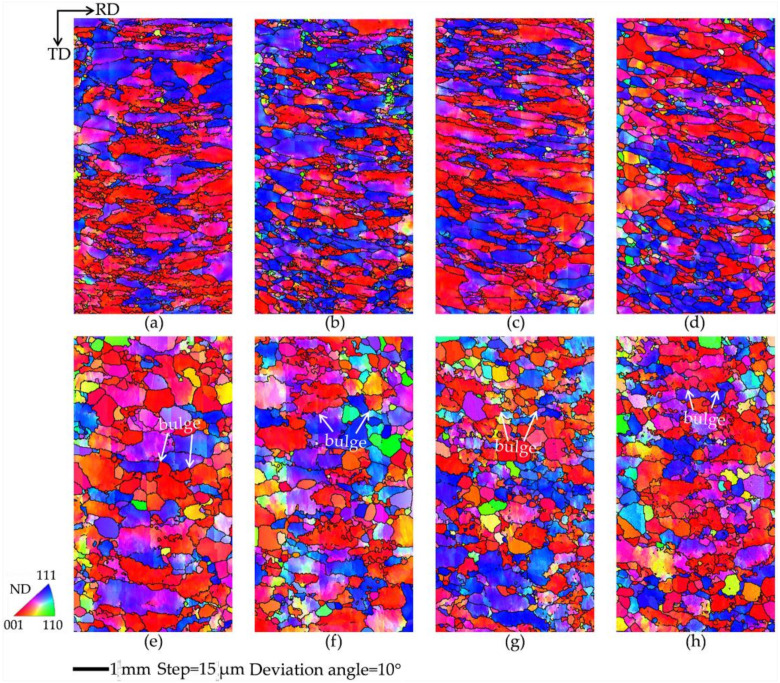
Microstructure under two deformation conditions, 900 °C with 5 s^−1^ (**a**–**d**) and 1150 °C with 1 s^−1^ (**e**–**h**), for four initial textures: (**a**,**e**) initial texture I, (**b**,**f**) initial texture II, (**c**,**g**) initial texture III, and (**d**,**h**) initial texture IV.

Figure 3 shows the microstructures reconstructed from the EBSD data under two hot deformation conditions. After deformation at 900 °C with 5 s^−1^, most of the grains are highly elongated, with straight grain boundaries along the RD direction without DRX. When deformed at 1150 °C with 1 s^−1^, many grain boundaries feature bulges which are dependent on the initial texture. Yang [9] investigated the microstructure evolution during the hot deformation of Ti-5Al-5Mo-5V-1Cr-1Fe alloy, and proposed SIBM to illustrate the bowed grain boundaries. Beladi [10] observed the bulges through the deformation course of Ni-30%Fe austenitic model alloy, and suggested that the phenomenon is due to SIBM. For initial texture I, a few λ grains bow out and small bulges form toward high-Taylor-factor grains. More and smaller bugles can be observed in some λ grains for initial texture II than initial texture I. For initial texture III, the λ grains have consumed many deformed grains with a high Taylor factor, and the bulges are larger than those of initial texture I. In initial texture IV, the λ grains have consumed a large amount of deformed grains with a high Taylor factor, resulting in the largest bulges among the four initial textures. Therefore, SIBM is the dominant DRX mechanism, and microstructure evolution is sensitively related to the initial texture.

**Figure 4:**
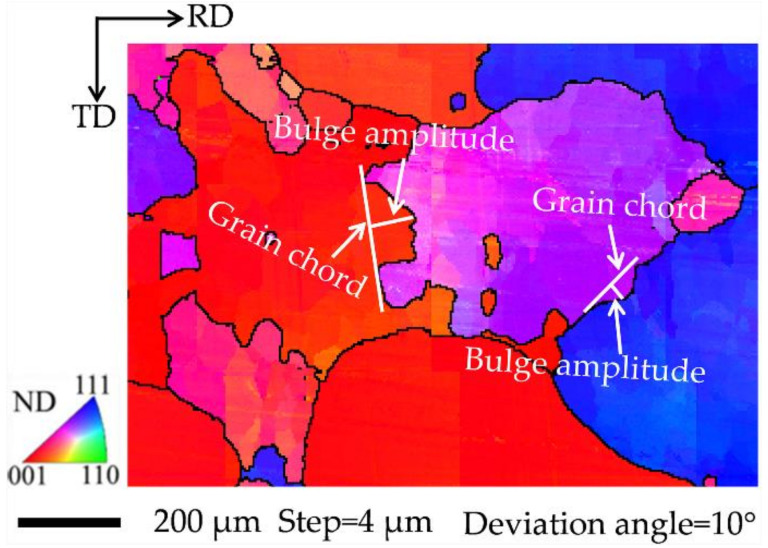
Local enlarged map of a region in Figure 3, indicating the definition of bulge amplitude.

Figure 4 illustrates the definition of bulge amplitude as the maximum length perpendicular to the grain chord [23]. Beladi [24] reported that the bulge amplitude of the grain boundary is similar to the DRX grain size based on SIBM. Unlike the microstructure close to the dies with a large grain size and little local misorientation due to the small strain, the microstructure in the center region consists of deformed grains and DRX grains. The average grain size of the deformed grains is large, while that of the DRX grains is small. The interior of the deformed grains evidences strain accumulation with higher local misorientaion and the presence of small strain-free DRX grains, whereas some coarser DRX grains which formed earlier exhibit higher local misorientation due to secondary deformation. Table 1 gives the statistical results of the grain boundary type for four initial textures under two hot deformation conditions. It is found that both the bulge fraction and amplitude, together with the fraction and size of equiaxed grains, present distinct differences among the different initial textures.

### 3.2. Texture Evolution

**Figure 5:**
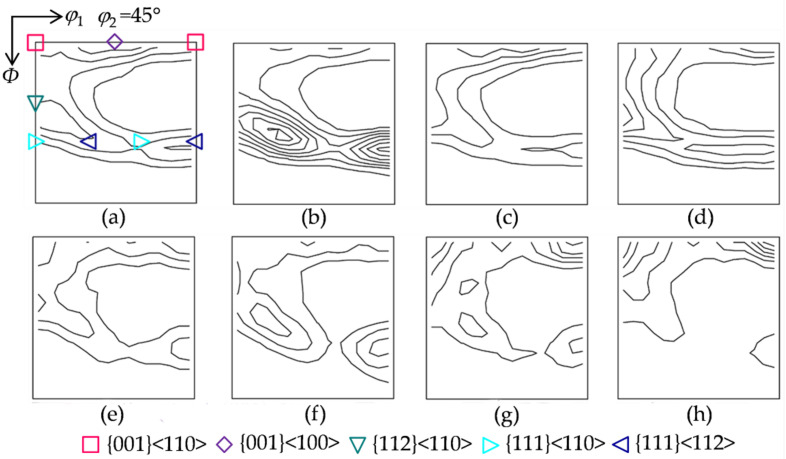
Constant *φ*_2_ = 45° section of the ODFs (levels: 1, 2, 3…) under two hot deformation conditions, 900 °C with 5 s^−1^ (**a**–**d**) and 1150 °C with 1 s^−1^ (**e**–**h**), for four initial textures: (**a**,**e**) initial texture I, (**b**,**f**) initial texture II, (**c**,**g**) initial texture III, and (**d**,**h**) initial texture IV.

Figure 5 presents the texture characteristics for four initial textures under two hot deformation conditions. After being compressed at 900 °C with 5 s^−1^, the deformation texture is composed of partial α fiber {111}–{001}<110> and complete λ and γ fibers, whereas the orientation density distribution varies with the initial texture. For initial texture I, the deformation texture consists of λ fiber with a peak at {001}<100>, α fiber with a peak at {112}<110>, and γ fiber with a peak at {111}<112>. For initial texture II, the γ fiber with a peak at {111}<112> is significantly enhanced as a major component, while the λ and α fibers are weakened. A similar texture occurs for initial textures III and I. For initial texture IV, α fiber with a peak at {112}<110> has the highest orientation density among the four initial textures, and the γ fiber is stronger than that in initial texture I. The texture is generally weakened at 1150 °C with 1 s^−1^, where γ fiber with a peak at {111}<112> has a higher orientation density for initial texture II, and {001}<110> exhibits a higher orientation density for initial textures IV and III. Generally, with the proceeding DRX, the low-Taylor-factor texture component ({001}<110>) is enhanced, and the high-Taylor-factor texture components ({111}<112> and {111}<110>) are weakened. Medium-Taylor-factor texture components ({001}<100> and {112}<110>) for different initial textures may experience an opposite variation.

### 3.3. DRX Texture by SIBM

**Figure 6:**
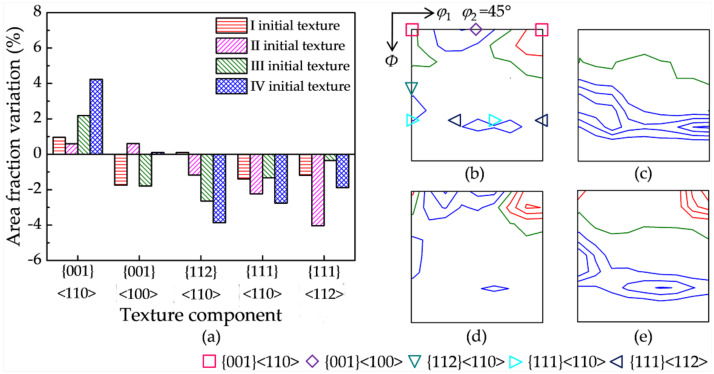
(**a**) Area fraction and orientation density variation (red line levels: 1, 2, 3…; olive line level: 0; blue line levels: −1, −2, −3…) of the main texture components for the four initial textures: (**b**) initial texture I, (**c**) initial texture II, (**d**) initial texture III, and (**e**) initial texture IV.

Hot deformation texture results from the combination of orientation rotation by plastic deformation and DRX by SIBM. The contribution of SIBM to the texture evolution is extracted by subtracting the deformation texture without DRX (900 °C with 5 s^−1^) from the hot deformation texture with DRX (1150 °C with 1 s^−1^). In order to identify the SIBM effect, Figure 6 shows the area fraction and orientation density variation of the main texture components by SIBM. {001}<110> exhibits the smallest and largest increase in the area fraction for initial textures II and IV, respectively. {001}<100> shows an obvious decrease for initial textures I and III, while it changes little for initial textures II and IV. {112}<110> exhibits a significant decrease for initial textures IV and III compared with initial textures II and I. {111}<110> shows an evident decrease, especially for initial textures IV and II. {111}<112> presents the largest and smallest decrease for initial textures II and III, respectively.

The critical orientation boundary separating the enhanced and weakened texture components (the olive lines in Figure 6) for initial textures Ι and III is the orientation line surrounding {001}<110>, and it is closer to {001}<110> for initial texture III. In contrast, the critical boundaries for initial textures II and IV are the orientation lines deviating about 25° and 30° from γ fiber, respectively. The area fraction variation of the main texture components and the shift of the critical orientation boundary indicate the sensitivity of the DRX texture by SIBM to the initial texture.

**Figure 7:**
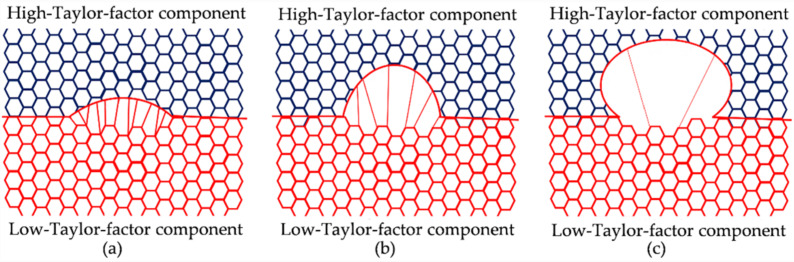
Schematic of the formation of SIBM with a boundary separating the high-Taylor-factor component and low-Taylor-factor component during hot deformation: (**a**) a bulge boundary forming with the dragging of the dislocation behind it, (**b**) a migrating bulge boundary accompanied by elongated dislocation with decreasing misorientation behind it, and (**c**) a DRX grain forming with few dislocations.

Based on the above analysis, the formation process of SIBM during hot deformation is identified, and the schematic diagram is shown in Figure 7. At the early stage of deformation, a bulge boundary toward a high-Taylor-factor component forms, and the dislocation structure behind it is elongated. With the proceeding deformation, the bulge boundary migrates continuously, while the density and misorientation of the elongated dislocation structure decrease. Finally, a DRX grain with few dislocation substructures forms.

## 4. Discussion

### 4.1. Critical Orientation Boundary

Based on the shift of the critical orientation boundary with the proceeding DRX, a quantitative relationship is required to represent the dependence of the critical Taylor factor on the texture. In order to cause a section of the grain boundary to bulge, the Taylor factor difference between adjacent grains must be higher than a critical value, which can be approximated as the subtraction of the statistically averaged Taylor factor and the critical Taylor factor. The Taylor factor (M) is defined as the ratio of the sum of the shear strain on various slip systems (dΓ) to the total normal strain imposed (${d\varepsilon }_{x}$) in a deformation step [1,20,25]:

(1) $$M=\frac{d\Gamma \;}{d\varepsilon_{x}}$$

Γ can be expressed as a relationship between the average moving distance (L) and the density (ρ) of dislocations [26]:

(2) Γ=ρbL

where b is Burger’s vector ($0.248{\;\times \;10}^{-9}\;m$) [26] and L is half of the grain diameter (*D*) adopted in the present study. Equations (1) and (2) can be combined to give the Taylor factor difference in terms of the dislocation density difference (∆ρ):

(3) $$\Delta M=\frac{bD\Delta \rho \;}{2\varepsilon_{x}}$$

The stored strain energy difference (∆E) with respect to ∆ρ is [26]

(4) $$\Delta E=\frac{Gb^{2}\Delta \rho \;}{2}$$

where *G* is the shear modulus. ΔE and the grain boundary energy ($\gamma_{s}$) contribute to the bulge of an (circular) area of the original boundary, and the criterion for the bulge in terms of the curvature (*R*) of the area free to migrate is given as

(5) $$\Delta G=-\Delta E+\frac{{2\gamma }_{s}}{R}$$

where ∆G is the difference of the Gibbs Free energy between two adjacent grains, and ∆G increases with the decreasing *R*. There are many significant differences in the *R* of bulges, and the *R* formed at the early stage of deformation is far away from *D*. However, the critical Taylor factor ($M_{0}$) is the critical maximum value for bulging, and the corresponding ∆G is close to 0. Thus, the ∆E of a bulging boundary can also be expressed by the grain boundary energy and curvature [26,27]:

(6) $$\Delta E=\frac{2\gamma_{s}\;}{R}$$

where *R* is close to *D*, $\gamma_{s}$ = ${617\times 10}^{-3}{\;\mathrm{Jm}}^{-2}$ [26] and G = ${47\times 10}^{9}\;\mathrm{Pa}$ [28]. Thus, the critical dislocation density difference is obtained by combining Equations (4) and (6):

(7) $$\Delta \rho =\frac{4\gamma_{s}\;}{{Gb}^{2}D}$$

According to Equations (3) and (7), the critical Taylor factor difference can be expressed in terms of $\gamma_{s}$ and $\varepsilon_{x}$:

(8) $$∆M_{c}=\frac{{2\gamma }_{s}}{{Gb\varepsilon }_{x}}$$

Therefore, the critical Taylor factor is written as

(9) $$M_{0}=\bar{M}-\frac{2\gamma_{s}\;}{Gb\varepsilon_{x}}$$

Here, $\bar{M}$ is the statistically averaged Taylor factor.

**Figure 8:**
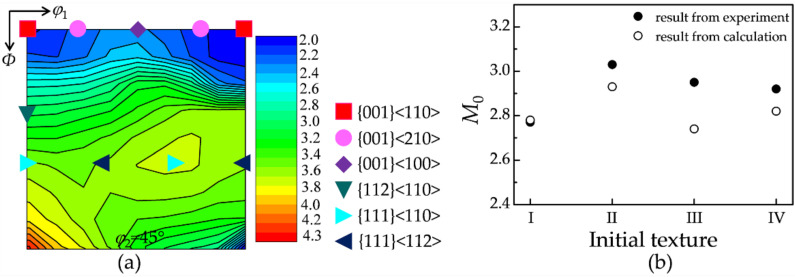
(**a**) A constant *φ*_2_ = 45° section of the Taylor factor distribution; (**b**) the calculated and measured $M_{0}$ values for the four initial textures.

Figure 8a shows the Taylor factor distribution on the crystal orientation, which is calculated using a full constraint Taylor model under plane strain. It is evident that the value of the Taylor factor depends sensitively on the texture component. Figure 8b illustrates the calculated $M_{0}$ values for four initial textures, which agree well with the experimental values in Section 3.3. Consequently, Equation (9) can efficiently describe the critical Taylor factor dependent on the instantaneous texture.

### 4.2. DRX Texture Model

The basic idea of the present model is inspired by the low-Taylor-factor nucleation model of Baczynski and Jonas [8], where the nucleation probability ($P_{g_{i}}^{N}$) of orientation $g_{i}$ with a Taylor factor lower than $M_{0}$ is written as

(10) $$P_{g_{i}}^{N}\;=\;\exp\left[-{\left(\frac{M_{g_{i}}-M_{\min}}{M_{0}-M_{\min}}\right)}^{n}\right]$$

Here, n is a Gaussian exponent, $M_{\min}$ is the minimum Taylor factor, and $M_{g_{i}}$ is the Taylor factor of orientation $g_{i}$. The texture evolution in the present study mainly results from SIBM, which is basically consistent with low-Taylor-factor nucleation. During the process of SIBM, the lower-Taylor-factor nucleus bulges to a larger amplitude. Thus, the model of Baczynski and Jonas can be used to calculate texture evolution by SIBM. Beladi [10] reported that the prominent DRX nucleation mechanism of a Ni-30%Fe austenitic model alloy is SIBM, and the texture evolution results from the preferred nucleation of the low-Taylor-factor component.

Variable n is applied to modulate the dependence of $P_{g_{i}}^{N}$ on the texture morphology. Here, the value of n is approximately defined as the ratio of the medium and statistically average Taylor factors:

(11) $$n=(\frac{M_{\max}{+M}_{\min}}{2})/\bar{M}$$

where $M_{\max}$ is the maximum Taylor factor. Thus, n=1 indicates the uniform orientation density distribution, while n≠1 means non-uniform orientation density distribution.

The nucleation probability, as well as the growth rate dependent on the Taylor factor difference between adjacent grains, evolves continuously with the texture. A quantitative model is then proposed to differentiate the evolution of various texture components, where the Taylor factors involved in the nucleation probability and growth rate are all employed as a variable. The volume fraction increment ($∆V_{g_{i}}$) of orientation $g_{i}$ with a Taylor factor lower than $M_{0}$ in one DRX step (3% DRX fraction) is written as

(12) $$∆V_{g_{i}}{=KP}_{g_{i}}^{N}S_{g_{i}}\left(\bar{M_{g_{i}}^{A}}-M_{g_{i}}\right)$$

where K is a constant, $S_{g_{i}}$ is the grain boundary area of orientation $g_{i}$, and $\bar{M_{g_{i}}^{A}}$ is the averaged Taylor factor of adjacent grains surrounding orientation $g_{i}$. If the grain size difference among low-Taylor-factor texture components is neglected, the proportion of the volume fraction increment of orientation $g_{i}$ ($f_{g_{i}}$) in the total increments of all of the low-Taylor-factor texture components in one DRX step is

(13) $$f_{g_{i}}{=P}_{g_{i}}^{N}V_{g_{i}}\left(\bar{M_{g_{i}}^{A}}-M_{g_{i}}\right)/\left(\sum_{i=1}^{l}P_{g_{i}}^{N}V_{g_{i}}\left(\bar{M_{g_{i}}^{A}}-M_{g_{i}}\right)\right)$$

where l is the number of texture components with a Taylor factor lower than $M_{0}$, and $V_{g_{i}}$ is the volume fraction of orientation $g_{i}$.

Similarly, the proportion of the volume fraction’s decrement of orientation $g_{j}$ ($f_{g_{j}}$) in the total decrements of all high-Taylor-factor texture components in one DRX step is

(14) $$f_{g_{j}}{=V}_{g_{j}}\left(M_{g_{j}}-\bar{M_{g_{j}}^{A}}\right)/\left(\sum_{j=1}^{m}V_{g_{j}}\left(M_{g_{j}}-\bar{M_{g_{j}}^{A}}\right)\right)$$

where m is the number of high-Taylor-factor texture components, $V_{g_{j}}$ is the volume fraction of orientation $g_{j}$, $M_{g_{j}}$ is the Taylor factor of orientation $g_{j}$, and $\bar{M_{g_{j}}^{A}}\;$ is the averaged Taylor factor of adjacent grains surrounding orientation $g_{j}$. From Equations (10) to (14), all of the parameters are renewed after each DRX step to capture the texture evolution by SIBM in the case of a different initial texture and hot deformation process.

### 4.3. DRX Texture Calculation

**Figure 9:**
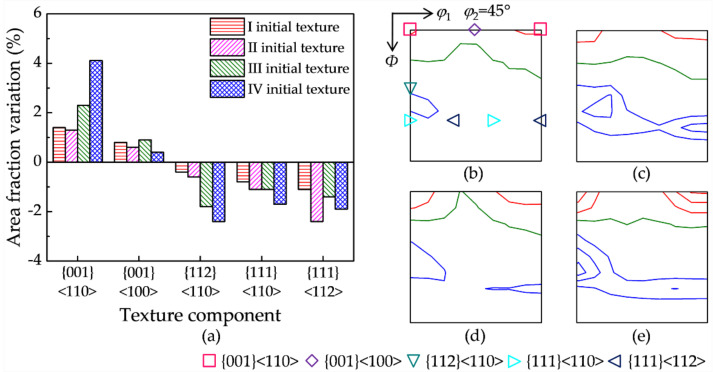
(**a**) The calculated area fraction and (**b**–**e**) orientation density variation (red line levels: 1, 2, 3…; olive line level: 0; blue line levels: −1, −2, −3…) of the main texture components between the DRX stage for four initial textures: (**b**) initial texture I, (**c**) initial texture II, (**d**) initial texture III, and (**e**) initial texture IV.

Figure 9 shows the calculated variation of the area fraction and orientation density of the main texture components between the DRX stage corresponding to two hot deformation parameters. {001}<110> has a more evident increase for initial texture IV, while {001}<100> has a very slight increase for all four initial textures. {112}<110> shows a larger decrease for initial textures IV and III. {111}<112> presents a larger decrease for initial textures II and IV, while {111}<110> shows a similar and moderate decrease for the four initial textures. The calculated variations of the main texture components are basically in good agreement with the EBSD measurement in Figure 6. Furthermore, the critical orientation boundaries are also successfully captured for the different initial textures.

Accordingly, the present method can quantitatively describe the DRX texture evolution by SIBM with regard to various initial textures. The accurate understanding and prediction of the texture evolution are highly valuable for the design and control of the hot deformation texture.

## 5. Conclusions

1. Dynamic recrystallization occurs during the high-temperature plane-strain compression of Fe-3.0 wt.% Si alloy by strain-induced boundary migration. The evolutions of the microstructure and texture, and the critical orientation boundary separating the weakened and enhanced texture components varies with the initial texture.
2. A quantitative relationship between the critical Taylor factor and the instantaneous texture is established, which is more reasonable than the traditional estimated constant value. A model is proposed to differentiate the evolution of various texture components by incorporating the nucleation probability and critical Taylor factor as a variable dependent on the orientation density distribution during hot deformation.
3. The calculated texture evolution and critical Taylor factor matches well with the experimental measurement for different initial textures, indicating the capability of the proposed method to predict and optimize the hot deformation texture produced by DRX based on SIBM as a function of the initial texture and dynamic process.

## Figures and Tables

**Table 1:** Grain boundary type for four initial textures under two hot deformation conditions in Fe-3.0 wt.% Si alloy.

Initial Texture	Deformation Parameters	Grain Boundary Type				
		Straight Boundary	Bulge Boundary		Equiaxed Grain Boundary	
		Fraction (%)	Fraction (%)	Average Amplitude (μm)	Fraction (%)	Average Grain Size (μm)
I	900 °C and 5 s^−1^	97	3	—	0	—
	1150 °C and 1 s^−1^	68	26	84	6	103
II	900 °C and 5 s^−1^	92	8	—	0	—
	1150 °C and1 s^−1^	71	22	78	7	98
III	900 °C and 5 s^−1^	97	3	—	0	—
	1150 °C and 1 s^−1^	63	27	89	10	107
IV	900 °C and 5 s^−1^	94	6	—	0	—
	1150 °C and 1 s^−1^	44	40	94	16	141

## Data Availability

The data is contained within the article.
